# Inhibition of p21 Activated Kinase (PAK) Reduces Airway Responsiveness In Vivo and In Vitro in Murine and Human Airways

**DOI:** 10.1371/journal.pone.0042601

**Published:** 2012-08-10

**Authors:** Wyn C. Hoover, Wenwu Zhang, Zhidong Xue, Huanling Gao, Jonathan Chernoff, D. Wade Clapp, Susan J. Gunst, Robert S. Tepper

**Affiliations:** 1 Department of Pediatrics, Indiana University School of Medicine, Indianapolis, Indiana, United States of America; 2 Herman B Wells Center for Pediatric Research, Indiana University School of Medicine, Indianapolis, Indiana, United States of America; 3 Department of Cellular and Integrative Physiology, Indiana University School of Medicine, Indianapolis, Indiana, United States of America; 4 Fox Chase Cancer Center, Philadelphia, Pennsylvania, United States of America; 5 Department of Microbiology and Immunology, Indiana University School of Medicine, Indianapolis, Indiana, United States of America; McMaster University, Canada

## Abstract

The p21-activated protein kinases (Paks) have been implicated in the regulation of smooth muscle contractility, but the physiologic effects of Pak activation on airway reactivity *in vivo* are unknown. A mouse model with a genetic deletion of Pak1 (*Pak1* ^−/−^) was used to determine the role of Pak in the response of the airways *in vivo* to challenge with inhaled or intravenous acetylcholine (ACh). Pulmonary resistance was measured in anesthetized mechanically ventilated *Pak1* ^−/−^ and wild type mice. *Pak1* ^−/−^ mice exhibited lower airway reactivity to ACh compared with wild type mice. Tracheal segments dissected from *Pak1* ^−/−^ mice and studied *in vitro* also exhibited reduced responsiveness to ACh compared with tracheas from wild type mice. Morphometric assessment and pulmonary function analysis revealed no differences in the structure of the airways or lung parenchyma, suggesting that that the reduced airway responsiveness did not result from structural abnormalities in the lungs or airways due to Pak1 deletion. Inhalation of the small molecule synthetic Pak1 inhibitor, IPA3, also significantly reduced in vivo airway responsiveness to ACh and 5-hydroxytryptamine (5-Ht) in wild type mice. IPA3 inhibited the contractility of isolated human bronchial tissues to ACh, confirming that this inhibitor is also effective in human airway smooth muscle tissue. The results demonstrate that Pak is a critical component of the contractile activation process in airway smooth muscle, and suggest that Pak inhibition could provide a novel strategy for reducing airway hyperresponsiveness.

## Introduction

Asthma is characterized by repeated episodes of reversible airway obstruction and airway hyperresponsiveness to non-specific stimuli. An effective approach for the reduction of airway hyperresponveness and obstruction has been to inhibit airway smooth muscle contraction using bronchodilators. While beta-adrenergic bronchodilators have been the primary therapy for decades; increasing concerns about the long term safety and efficacy of these agents have led to a need for novel approaches to reduce airway smooth muscle responsiveness.

The p21-activated protein kinases (Paks) have been implicated in the regulation of cell motility and contractility in many eukaryotic cell types. We therefore hypothesized that Pak might provide a novel target for the reduction of airway hyperresponsiveness [1]–[5]. Although Pak has been previously implicated in the regulation of smooth muscle contractility, the physiologic effects of Pak activation on airway reactivity in vivo are unknown [6]–[8].

Pak 1, 2 an 3 isoforms are expressed in airway smooth muscle and have been implicated in the regulation of cytoskeletal dynamics in multiple cell types [9], [10]. Pak1 is implicated in the regulation of smooth muscle cell motility and contraction and has been described as the dominant isoform in smooth muscle tissues [1], [8], [11]. In the present study, we used a mouse model with a genetic deletion of Pak1 to determine whether Pak regulates airway reactivity under physiologic conditions, and whether it could provide a target for the suppression of airway responsiveness [12]. We also tested the effects of a synthetic small molecule Pak inhibitor, IPA3, on airway reactivity *in vivo* [13]. IPA-3 is an allosteric Pak inhibitor that specifically inhibits the auto-activation of Pak isoforms 1, 2 and 3 [13], [14]. In addition, we evaluated the effects of Pak inhibition on the responsiveness of murine and human airway smooth muscle tissues *in vitro*.

Our findings demonstrate that the suppression of Pak activity reduces airway responsiveness, and suggest that Pak could be an important molecular target for the suppression of airway reactivity *in vivo*.

## Results

### Expression of Pak1 isoform is inhibited in airway tissues from *Pak1−/−* mice

**Figure 1:**
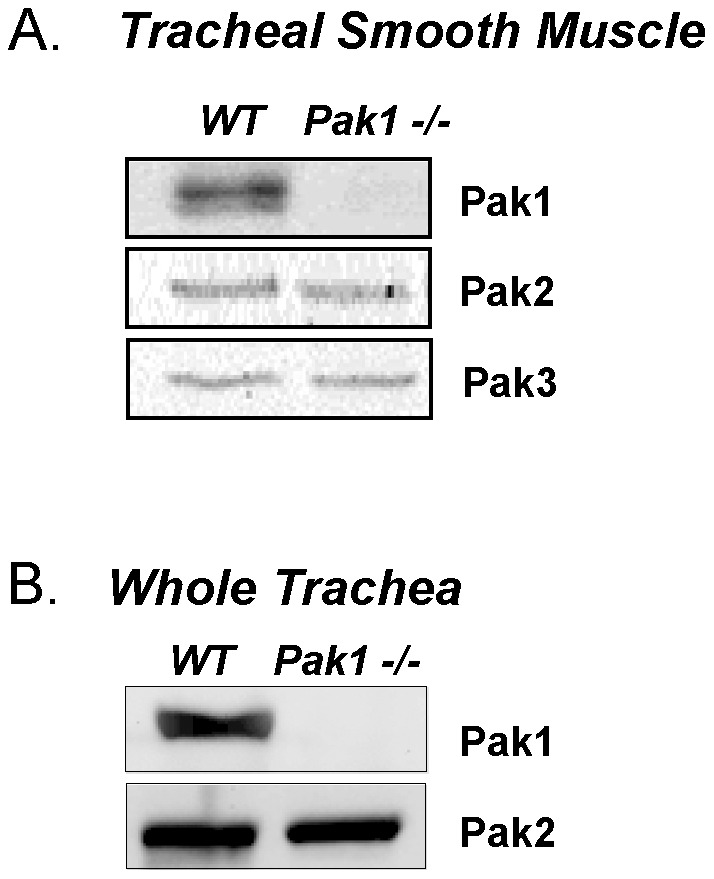
Pak1, Pak2 and Pak3 isoforms were detected in WT murine tracheal smooth muscle by immunoblot. No Pak1 was detected in extracts of isolated tracheal smooth muscle (A) or whole tracheas (B) from *Pak1* ^−/−^ mice. Immunoblots of tracheal smooth muscle were obtained from tracheal smooth muscle extracts pooled from 3 mice of each type. Whole trachea extracts were each from a single mouse.

We evaluated the expression of Pak1, Pak2 and Pak3 in WT and *Pak1^−/−^* murine tracheas and isolated murine airway smooth muscle by immunoblot. Pak1 was detected in extracts from isolated tracheal smooth muscle tissues and whole tracheas from WT mice, but not in *Pak1* ^−/−^ mice (Figure 1A, B). Pak2 and Pak3 isoforms were also expressed at similar levels in tracheal smooth muscle from both WT and *Pak1^−/−^* mice, indicating that the expression of Pak2 and Pak3 were not altered in the *Pak1* knockout mice (Figure 1A).

### Genetic Deletion of *Pak1* suppresses Pulmonary Responsiveness *in vivo*

**Figure 2:**
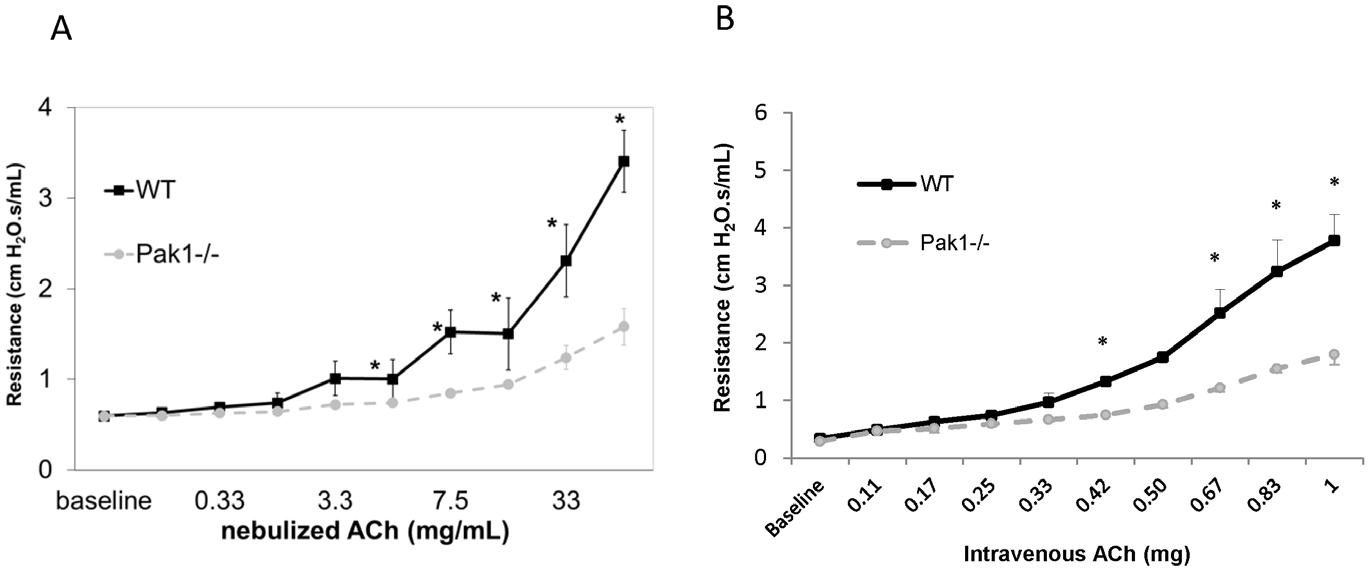
*In vivo* airway reactivity of Pak1*^−/−^* mice was lower than that of WT mice to aerosolized (A) and intra-venous (B) acetylcholine. Resistance in response to increasing concentrations of aerosolized acetylcholine (ACh) for wild-type (WT; N = 8) and Pak1*^−/−^* mice (N = 8) (A); values are means ± SEM. There was no difference in resistance at baseline. When analyzed by repeated measures ANOVA, resistance increased with increasing ACh dose (p<0.0001), Pak1*^−/−^* mice had a significantly smaller slope of the dose response curve (p<0.03), and a significantly smaller increase in resistance compared to WT mice (p<0.03). Post-hoc analysis demonstrated Pak 1*^−/−^* compared to WT mice had significantly smaller resistances at all ACh concentrations ≥7.5 mg/ml (p<0.05). Resistance in response to increasing concentrations of intravenous acetylcholine (ACh) for wild-type (WT; N = 4) and Pak1*^−/−^* mice (N = 4) (B); values are means ± SEM. There was no difference in resistance at baseline. When analyzed by repeated measures ANOVA, resistance for Pak1*^−/−^* mice increased less with increasing ACh dose (p<0.0001) compared to WT mice. Post-hoc analysis demonstrated Pak1*^−/−^* compared to WT mice had significantly lower resistances at ACh concentrations ≥0.42 mg (p<0.05).

We evaluated the role of Pak1 in the regulation of changes in pulmonary resistance in response to ACh in vivo by comparing responses of WT and *Pak1* ^−/−^ mice. Dose-response curves depicting increases in pulmonary resistance above baseline for increasing concentrations of inhaled ACh are illustrated in Figure 2A. There were no significant differences in the baseline resistances for either group. However, aerosolized ACh elicited significantly smaller increases in pulmonary resistance in *Pak1* ^−/−^ mice compared to WT mice (*P*<0.05).

We also evaluated the effect of the route of agonist delivery upon airway responsiveness by performing dose-response curves in response to intravenous challenge with ACh (Figure 2B). Again, there were no significant differences for baseline resistances in animals challenged with intravenous ACh, and ACh elicited significantly smaller increases in pulmonary resistance in *Pak1* ^−/−^ mice compared to WT mice (*P*<0.05). The fact that the similar results were obtained using intravenous versus aerosolized ACh indicates the effect of Pak deletion on airway reactivity cannot be attributed to alterations in the epithelial barrier function in Pak1−/− mice.

### Pharmacologic inhibition of Pak suppresses Pulmonary Responsiveness *In Vivo*

**Figure 3:**
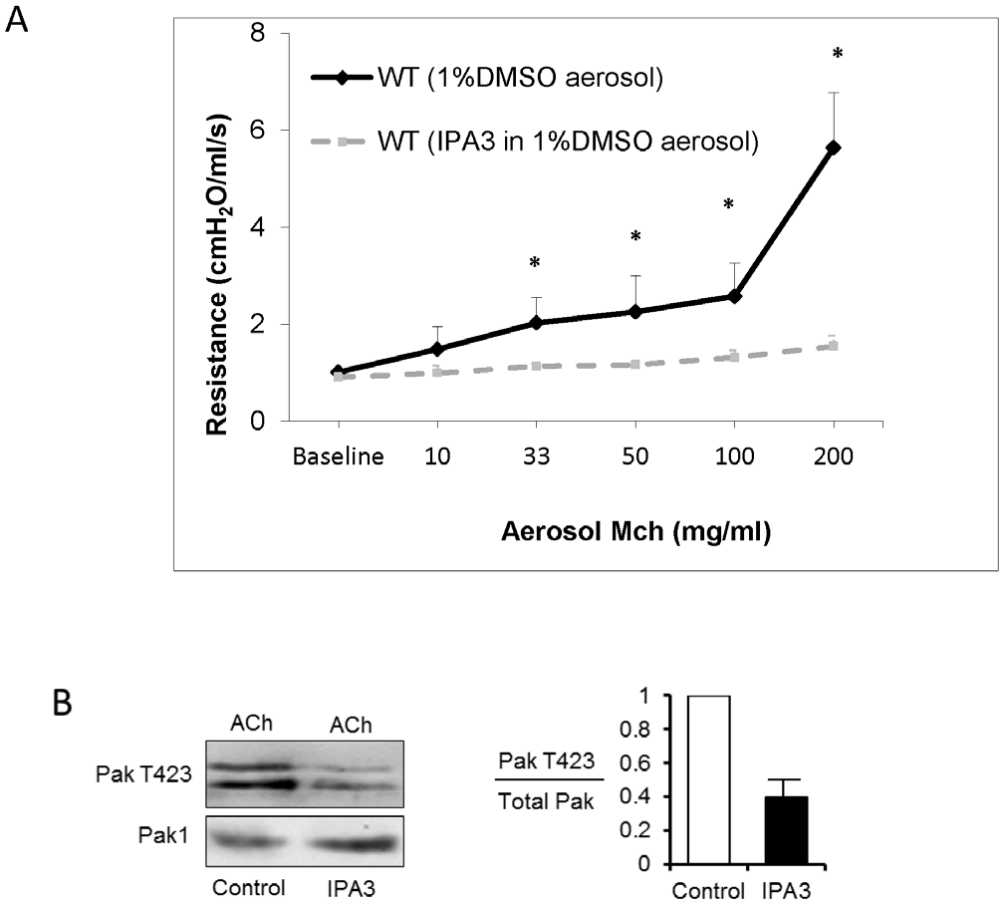
Aerosolized IPA3 inhibited airway contractility *in vivo* and suppressed Pak activation. When assessed by repeated ANOVA, resistance increased with increasing ACh dose (p<0.0001), and IPA3 dissolved in 1% DMSO (N = 3) and aerosolized 1-hour prior to bronchial challenge of WT mice significantly reduced the slope of the increase in resistance (p<0.0001), as well as the magnitude of the increase in resistance compared to control vehicle (1%DMSO; N = 5) (p<0.001) (A). Post-hoc analysis indicated that IPA3 treatment resulted in lower resistances at MCh doses ≥33 mg/ml (p<0.05). Tracheal smooth muscle isolated from WT mice treated *in vivo* with IPA3 demonstrated significantly lower Pak activation as assessed by Pak T423 phosphorylation following stimulation with ACh compared to airway smooth muscle isolated from WT mice (B). Results represent 2 samples of tracheal smooth muscle from each group. Each sample consisted of pooled tracheal muscle tissues from 3 separate mice with the same treatment.

We evaluated the effect of a small molecule synthetic Pak inhibitor (IPA3) on pulmonary reactivity to ACh in WT mice *in vivo*. IPA3 allosterically inhibits the catalytic activity of Pak1, 2 and 3 isoforms [11], [12]. Pak inhibitor IPA3 (20 µl of 5 mM, 1%DMSO) or Vehicle (1% DMSO) was nebulized into the ventilator circuit 60 minutes prior to bronchial challenge with aerosolized ACh. The respiratory response to inhaled ACh of WT mice receiving IPA3 was significantly decreased compared to that of WT mice that received Vehicle alone (Figure 3A).

In separate series of experiments, tracheas were isolated from WT animals that had received nebulized IPA3 *in vivo* to confirm the inhibition of Pak activation by IPA3. Phosphorylation of Thr 423 in the Pak catalytic domain occurs with its activation and is critical for its full catalytic function toward exogenous substrates [15], [16]. Following removal of the epithelium from isolated tracheas, immunoblots were performed on tracheal extracts to measure the phosphorylation of Pak on tyrosine 423. ACh elicited a smaller increase in Pak phosphorylation at Thr 423 in tracheal smooth muscle from IPA3-treated animals than in tracheas from WT animals, indicating that the dose of aerosolized IPA3 was effective at inhibiting Pak activation in vivo (Figure 3B).

### Genetic Deletion of *Pak1* Suppresses the Contractility of isolated Tracheal segments *in vitro*

**Figure 4:**
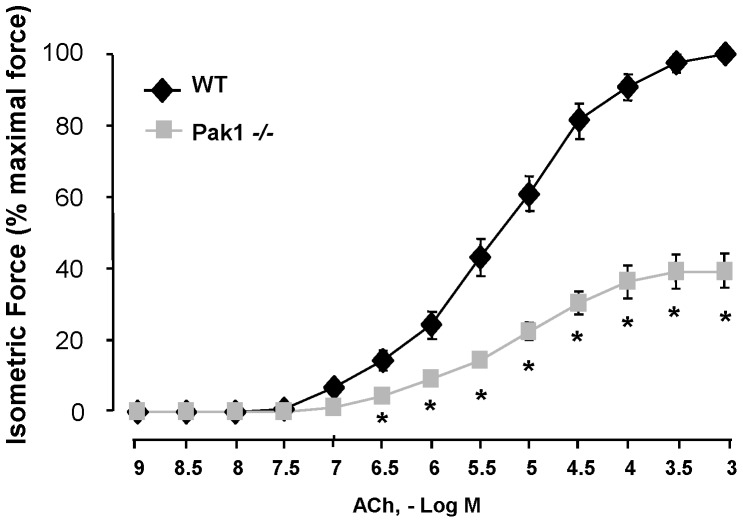
Tracheas isolated from *Pak1* ^−/−^ mice showed reduced contractility to ACh in vitro. Isometric force generation (% of maximal force to ACh stimulation in WT mice) was significantly lower in tracheas isolated from *Pak1* ^−/−^ (grey squares) mice compared to WT (black diamonds) mice (N = 10 or 11 in each group, p<0.01).

We evaluated the in vitro contractility of tracheal segments isolated from *Pak1−/−* mice. Tracheal segments from WT and *Pak1* ^−/−^ mice were isolated, the epithelium removed, and the segments suspended in organ chambers for the measurement of isometric contractile force in response to increasing concentrations of ACh. Tracheal segments from *Pak1* ^−/−^ mice developed significantly lower forces in response to ACh compared to tracheas from WT mice (*P*<0.05) (Figure 4).

### Genetic Deletion of *Pak1* does not affect lung parenchymal structure or function

**Figure 5:**
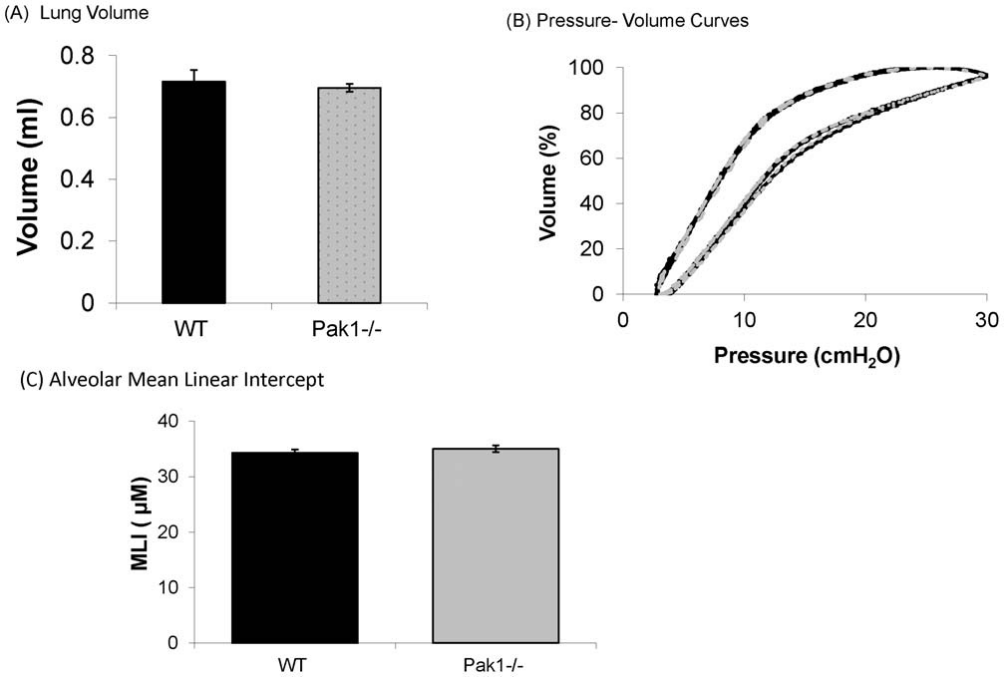
Comparison of the Lung Parenchyma (N = 3–5 in each group) for WT (black) and Pak1^−/−^ (grey) mice. There were no significant differences for lung volumes at 30 cmH_2_O (p>0.37) (A); pressure volume curves normalized to volume at 30 cmH_2_O (B); or Alveolar Mean Linear Intercepts (MLI) (p>0.40) (C).

The effect of *Pak1* deletion on the elastic properties of the lungs was evaluated by comparing pressure-volume curves obtained in WT and *Pak1* ^−/−^ animals in vivo. Mechanical ventilation was interrupted and the pulmonary system was slowly cycled between the end-expiratory pressure of 2.5 cm H_2_O and an airway pressure of 30 cm H_2_O. There were no significant differences in lung volume at 30 cm H_2_O between WT and *Pak1* ^−/−^ mice. There were also no differences in the pressure volume curves of the WT and Pak1^−/−^ animals (Figures 5A, B).

Alveolar size was compared in lungs from WT and *Pak1* ^−/−^ animals by measuring mean linear intercepts from histological sections taken from lungs that had been formalin fixed at 30 cm H_2_O. There were no significant differences in mean linear intercept for WT and *Pak1* ^−/−^ animals, indicating no differences in alveolar size (Figure 5C).

### Genetic deletion of *Pak1* does not affect airway structure

**Figure 6:**
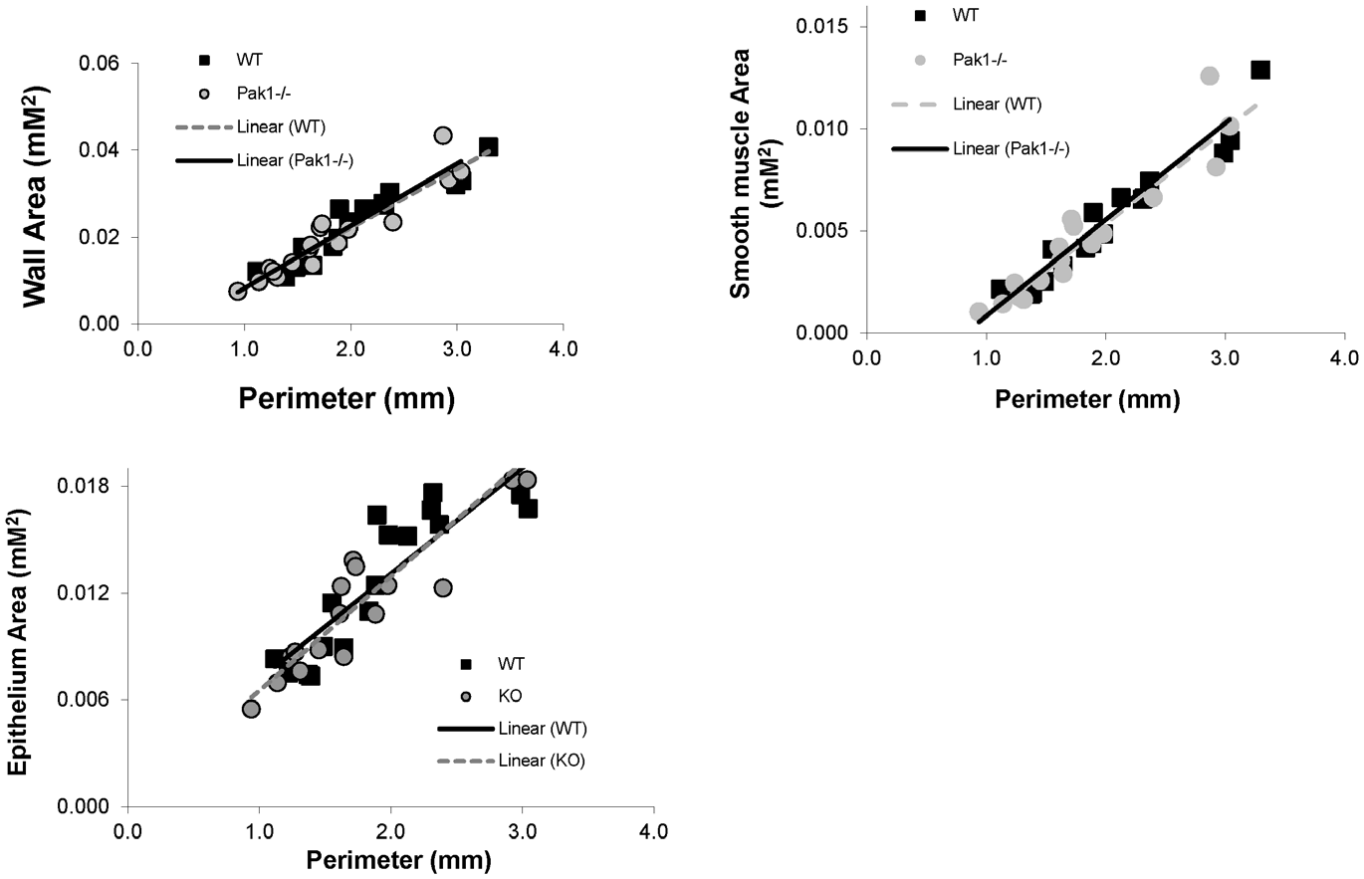
Comparison of Lung Histology for WT and *Pak1* ^−/−^ mice. Airway wall area (A), airway smooth muscle area (B), and airway epithelium area (C) were not significantly different between the WT (black squares) and *Pak1* ^−/−^ (grey circles) mice when assessed by ANOVA adjusting for airway perimeter (p>0.15). (N = 5 mice in each group).

The effects of *Pak1* deletion on the structure of the airways were evaluated by morphometric assessments of histologic sections of lungs from WT and *Pak1* ^−/−^ mice. There were no significant differences in the area of the airway wall or the areas of smooth muscle and epithelium (Figure 6).

### Pharmacologic inhibition of Pak inhibits Responsiveness of Murine Tracheal Segments *in vitro*

**Figure 7:**
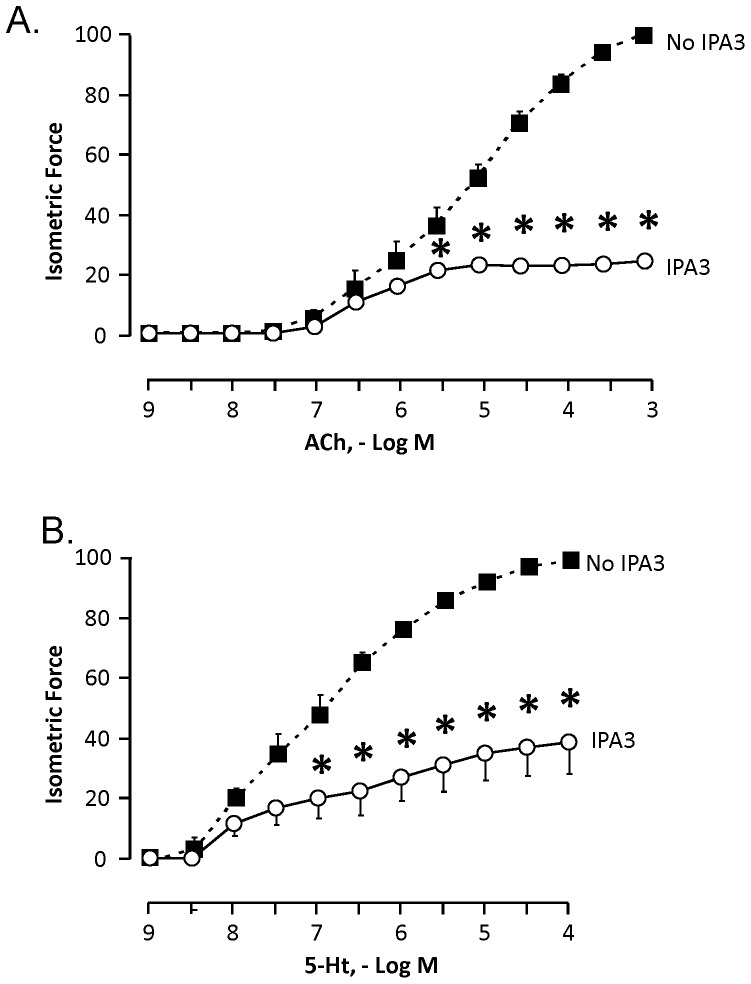
Pak inhibitor, IPA3, reduced the contractility of isolated murine tracheal segments to ACh and 5-Ht *in vitro*. Curves show responses of tracheal segments normalized to maximal response to ACh or 5-Ht prior to incubation with vehicle or IPA3. IPA3 treatment (open circles) significantly inhibited contractile responses to *(A)* ACh *(n = 4)* and *(B)* 5-Ht (n = 4) compared to responses of vehicle-treated tissues (black squares). Values are means ± SEM, significantly different, * p<0.05.

The effect of IPA3 on *the* contractility of murine tracheal segments to ACh was evaluated by concurrently stimulating pairs of tracheas with cumulative concentrations of acetylcholine (ACh) (10^−9^ to 10^−3^ M) or 5-hydroxytryptamine (5-Ht) (10^−9^ to 10^−4^) and then measuring the isometric contractile force at each concentration. The ACh or 5-Ht was then washed out and one trachea from each pair was treated with 100 µM IPA3 to inhibit PAK. The other trachea was treated with vehicle (1% DMSO). Tracheal segments were incubated in IPA3 or vehicle for 2 hours, after which a dose responsive curve to ACh or 5-Ht was repeated. The contractile responses to ACh and 5-Ht were significantly lower in tracheas treated with IPA3 compared to tracheas treated with vehicle (Figure 7).

### Pharmacologic inhibition of Pak inhibits Responsiveness of Human Bronchial Rings *in vitro*

**Figure 8:**
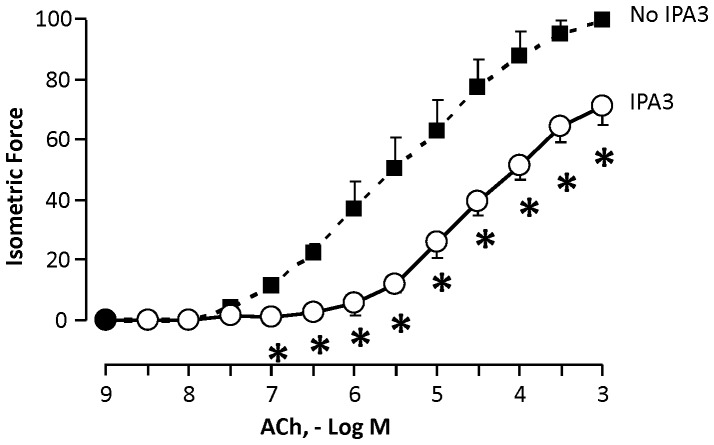
Pak inhibitor, IPA3, reduced the contractility of isolated human bronchial segments to ACh *in vitro*. Curves show responses of bronchial rings normalized to maximal ACh response obtained prior to incubation with vehicle or IPA3. IPA3 treatment (open circles) significantly inhibited contractile responses to ACh compared to responses of vehicle-treated bronchi (black squares). Values are means ± SEM, (n = 3 pairs of bronchi), * p<0.05.

The effect of IPA3 on *the* contractility of human bronchial rings to ACh was evaluated by concurrently stimulating pairs of bronchial rings with cumulative concentrations of acetylcholine (ACh) (10^−9^ to 10^−3^ M) as described above. The contractile responses to ACh were significantly lower in bronchi treated with IPA3 compared to bronchi treated with vehicle (Figure 8). The inhibition of PAK activation by IPA3 was confirmed by western blot for PAK T423 phosphorylation (data not shown).

## Discussion

Our results demonstrate that inhibiting the activity of p21-activated protein kinase is an effective approach for reducing airway responsiveness *in vivo*. Mice with a genetic deletion of Pak1 exhibited significantly lower airway reactivity to acetylcholine *in vivo*, and the aerosol administration of a synthetic small molecule Pak inhibitor also reduced airway responsiveness in mice *in vivo*. Furthermore, isolated tracheal segments from *Pak1^−/−^* mice exhibited reduced contractility to ACh *in vitro*, indicating that the decrease in airway reactivity observed *in vivo* in *Pak1^−/−^* mice reflected a decrease in force generation by the airway smooth muscle. As no differences in the structure of the airways and lung parenchyma were detected in *Pak1* ^−/−^ and WT animals, decreased airway responsiveness did not appear to result from other factors that can alter airway responsiveness. Taken in sum, our observations provide strong evidence that the reduction of Pak activity lowers airway responsiveness *in vivo*, and that this effect is caused by a reduction in the contractility of the airway smooth muscle. The fact that Pak inhibition was also effective in reducing airway smooth muscle responsiveness in human bronchial tissues, which suggests that this approach could be effective in humans *in vivo* also.

We could not detect any differences in the structure or physical properties of the lungs from *Pak1^−/−^* mice as compared to WT mice. The quantity of airway smooth muscle within the airway wall was the same in the WT and *Pak1* ^−/−^ mice and airway morphometry was similar. There were also no differences between WT and *Pak1* ^−/−^ mice in lung parenchymal structure or function. These results suggest the lower level of airway reactivity in *Pak1^−/−^* mice are unlikely to be due to differences in lung structure. Previous studies that have evaluated the functions of other organ systems in *Pak1* ^−/−^ mice have also not detected major developmental abnormalities or organ defects [12], [17], [18]. Taken in sum, our findings in the *Pak1^−/−^* mice provide strong evidence that their decreased airway responsiveness *in vivo* results from the absence of Pak1 in their airway smooth muscle tissue, and that this reflects a critical role for Pak1 in the contractile activation of airway smooth muscle.

We showed that the synthetic Pak inhibitor, IPA3, was effective at inhibiting ACh-induced Pak activation in airway tissues both *in vivo* and *in vitro*. We also showed that IPA3 inhibits the contractile response of airway tissues to 5-Ht *in vitro*, indicating that the effects of IPA3 are not agonist-specific. IPA3 is an allosteric Pak inhibitor that binds outside the ATP-binding pocket and specifically inhibits the auto-activation of Pak isoforms 1, 2 and 3 [13], [14]. Treatment of WT mice with IPA3 inhibited airway responsiveness *in vivo* and *in vitro* similarly to that observed in the *Pak1* ^−/−^ mice. These results provide further support for our conclusion that the reduced airway responsiveness in *Pak1* ^−/−^ mice results from the loss of Pak1 activation in airway smooth muscle in response to agonist stimulation, rather than from an unidentified developmental defect caused by the absence of Pak1. IPA3 also inhibited the contractility of isolated human bronchial tissues to ACh, confirming that this inhibitor is also effective in human airway smooth muscle tissue.

This is the first study to demonstrate a role for Pak1 in the regulation of airway responsiveness *in vivo*. Previous studies that have evaluated the role of Pak in the regulation of smooth muscle contractile proteins have yielded inconsistent findings. Some of these studies have shown an inhibitory effect of Pak on contractile protein activation, suggesting that it would inhibit smooth muscle contractility [9], [17], whereas other studies indicate that Pak may contribute to the activation of contractile proteins and active tension development [8], [12], [13], [19]. Most of these studies were performed using Triton skinned muscle tissues and exogenous recombinant Pak [5]–[7], [16] or by expressing recombinant Pak in cultured cells [20]. The observations obtained using these preparations may not reflect the function of endogenous Pak in intact smooth muscle tissues under physiologic conditions. The results of our current studies evaluating airway responsiveness under physiologic conditions *in vivo* and *in vitro* clearly demonstrate that Pak is a critical intermediate in the contractile activation of airway smooth muscle and that its inhibition reduces airway responsiveness.

In conclusion, our findings demonstrate that Pak is an effective molecular target for inhibiting airway smooth muscle contraction and suppressing airway reactivity *in vivo*. This suggests that Pak inhibition could provide a novel strategy for reducing airway hyperresponsiveness in diseases such as asthma.

## Materials and Methods

### Ethics Statement

The protocols for these studies were approved by the Indiana University Institutional Animal Care and Use Committee (IACUC) and Indiana University Institutional Review Board, which waived the need for written consent for the use of surgical waste material for our research study and the tissue was, and remained de-identified.

### Animals

*Pak1* ^−/−^ mice on a C57BL/6 background were backcrossed 6 generations as previously described [10], [15]. *Pak1* ^−/−^ mice and Wild type (WT) C57BL/6 mice were studied at 8 weeks of age.

### Human Airway Tissues

Fresh non-identified human lung tissue from surgical waste was collected within 1–2 hours of removal from the lungs and preserved in physiologic saline solution. Tissues were studied *in vitro* immediately after they were obtained.

### *In Vivo* Pulmonary Measurements

WT and *Pak1^−/−^* mice were anesthetized with pentobarbital (6 mg/100 gm intra-peritoneal). Following tracheotomy, an 18- gauge cannula was inserted and securely tied with 4.0 braided silk. The animals were then mechanically ventilated with a computer-controlled small-animal ventilator (FlexiVent, SCIREQ, Montreal, Quebec) using a tidal volume of 8 mL/kg at a rate of 180 breaths/min and PEEP of 3.5 cm H_2_0. Pulmonary resistance was measured by the computer controlled ventilator by interrupting ventilation and imposing a 2.5 Hz sinusoidal signal and then resuming ventilation.

### Airway Challenge

Following measurement of baseline resistance, animals underwent airway challenge with normal saline and then increasing concentrations of acetylcholine (ACh) or methacholine (MCh) by either aerosol challenge or intravenous challenge. For aerosol challenge, 20 µL of each solution of ACh ranging from 0.3 to 50 mg/ml was nebulized in line with the ventilation circuit over 10 seconds. Resistance was then measured every 30 seconds for the next 4 minutes to record a maximal response for each ACh dose. For intravenous Challenge, increasing volumes (3–100 µL) of acetylcholine (10 mg/ml) were infused through a jugular vein catheter for 20 seconds to deliver Ach doses ranging from 0.03–1.0 mg. Measurements of resistance were obtained every 10 seconds for a minute and then the next Ach dose was administered.

### Evaluation of PAK inhibitor *in vivo* (IPA3)

The effects of the small molecule Pak inhibitor IPA3 [13] on in vivo pulmonary responsiveness was evaluated in WT animals. IPA3 (5 mM) was dissolved in 1% DMSO and 20 µl was nebulized into the ventilator circuit 60 minutes prior to performing the Ach dose response curves. Control animals received nebulized 1% DMSO prior to bronchial challenge.

### Pressure-volume curves of the lungs

The pulmonary elastic properties were evaluated from quasi-static pressure-volume curves obtained in vivo. Mechanical ventilation of the anesthetized animals was interrupted and the pulmonary system was slowly cycled between the end-expiratory pressure of 2.5 cm H2O and a maximal airway pressure of 30 cmH_2_O.

### Measurement of Contractility of Isolated Murine Tracheal Segments and Human Bronchial Rings *In Vitro*

*Pak1^−/−^* and WT mice were euthanized with pentobarbital (12 mg/100 g intra-peritoneal) and intact tracheal segments were removed and dissected free of the epithelium. The isolated tracheal segments were mounted on wires and attached to Grass force transducers in tissue baths for the measurement of isometric force in response to increasing doses of ACh.

In some experiments, tracheas were isolated from WT mice to evaluate the effect of Pak inhibition on the contractile responses of airway tissues. Dose-response curves to ACh (10^−9^ to 10^−3^ M) or 5-hydroxytryptamine (5-Ht) (10^−9^ to 10^−4^ M) were performed after 30 min incubation in physiologic saline. After washing the agonists from the tissues, one of each pair of the tracheal segments was treated with 100 µM IPA3 dissolved in 1% DMSO for 2 hours and the other segment was treated with 1% DMSO alone. A dose-response curve to ACh or 5-HT was then repeated on each pair of tissues concurrently.

Human bronchial rings (5 mm diameter, 2 mm wide) were dissected from lung specimens and cleaned of connective tissue. Rings were attached to force transducers as described above and equilibrated in a tissue bath for 30–60 min. Two rings were obtained from each lung specimen and studied concurrently. Dose-response curves to ACh (10^−9^ to 10^−3^ M) were performed after 30 min incubation in physiologic saline. After washing ACh from the tissue, one of the bronchial rings was treated with 100 µM IPA3 dissolved in 1% DMSO for 2 hours and the other ring was treated with 1% DMSO alone. A dose-response curve to ACh was then repeated on each tissue concurrently. Tissues from each group were quick frozen in liquid nitrogen for biochemical analysis to confirm the inhibition of Pak activation by IPA3.

### Analysis of protein expression and phosphorylation

Proteins were extracted from pulverized murine tracheal smooth muscle, whole murine tracheal segments or human bronchial rings. Tissue extracts were then boiled in sample buffer (1.5% dithiothreitol, 2% SDS, 80 mM Tris-HCl (pH 6.8), 10% glycerol and 0.01% Bromophenol Blue) for 5 min, separated using SDS polyacrylamide gel electrophoresis (SDS-PAGE), and transferred to nitrocellulose. Membranes were probed with antibodies followed by horseradish peroxidase (HRP)-conjugated secondary antibodies (Ig) (Jackson, Immunoresearch Co.). Proteins were visualized by enhanced chemiluminescence (ECL). Antibodies against Pak1, Pak2, Pak3 and Pak Thr423 were obtained from Cell Signaling.

### Lung and Airway Histology

Lung were excised and fixed with 10% formalin at a distending pressure of 20 cm H_2_O, sectioned into blocks, embedded in paraffin, and a 5-µm-thick section were stained with Masson's Trichrome. Tissues were visualized by light microscopy and images captured with a digital camera (SPOT, Diagnostic Instruments, Inc, MI), and morphometric measurements from the fixed tissues were obtained from the digital images using imaging software (Metamorph, version 5.01r, Universal Imaging). For airways, the internal perimeters were calculated from the luminal border of the airway epithelium. The cross-sectional areas of the airway wall, airway smooth muscle and epithelium were determined by point counting, as previously described [21]. For the parenchyma, alveolar size was quantified by measuring the mean linear intercept, which is the number of alveolar walls intersecting a line of known length [22], [23].
